# *catena*-Poly[[[dichloridozinc(II)]-μ-1,4-bis­(1*H*-benzimidazol-2-yl-κ*N* ^3^)butane] 1,4-bis­(1*H*-benzimidazol-2-yl)butane solvate]

**DOI:** 10.1107/S1600536809052635

**Published:** 2009-12-12

**Authors:** Yan-Ling Zhou, Ming-Hua Zeng, Seik Weng Ng

**Affiliations:** a School of Chemistry and Chemical Engineering, Guangxi Normal University, 541004 Guilin, People’s Republic of China; b Department of Chemistry, University of Malaya, 50603 Kuala Lumpur, Malaysia

## Abstract

In the crystal structure of the title coordination polymer/co-crystal, {[ZnCl_2_(C_18_H_18_N_4_)]·C_18_H_18_N_4_}_*n*_, the tetrahedrally coordinated Zn^II^ ions are linked by the *N*-heterocycle into a linear chain. Another *N*-heterocycle present is not coordinated to the metal atom but inter­acts with the chain through N—H⋯N and N—H⋯Cl hydrogen bonds. The butyl chain of the uncoordinated ligand is disordered over three positions in a 0.511 (4):0.289 (5):0.200 (5) ratio.

## Related literature

For the synthesis of the ligand, see: van Aldaba *et al.* (1995 ▶). For other metal(II) dichloride adducts of this *N*-heterocycle, see: Chen *et al.* (2005 ▶); Wang *et al.* (2006 ▶).
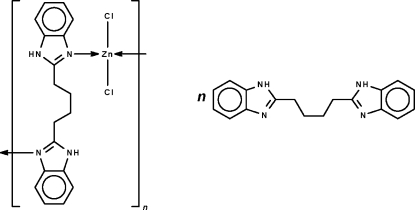


## Experimental

### 

#### Crystal data

[ZnCl_2_(C_18_H_18_N_4_)]·C_18_H_18_N_4_
*M* *_r_* = 717.00
Monoclinic, 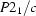

*a* = 8.5321 (5) Å
*b* = 24.119 (2) Å
*c* = 16.880 (1) Å
β = 92.999 (1)°
*V* = 3468.9 (4) Å^3^
*Z* = 4
Mo *K*α radiation
μ = 0.90 mm^−1^
*T* = 173 K
0.45 × 0.30 × 0.20 mm

#### Data collection

Bruker APEXII area-detector diffractometer
Absorption correction: multi-scan (*SADABS*; Sheldrick, 1996 ▶) *T*_min_ = 0.687, *T*_max_ = 0.840
17738 measured reflections
7549 independent reflections
3855 reflections with *I* > 2σ(*I*)
*R*_int_ = 0.052

#### Refinement

*R*[*F* ^2^ > 2σ(*F* ^2^)] = 0.062
*wR*(*F* ^2^) = 0.192
*S* = 1.02
7549 reflections
448 parameters
32 restraints
H atoms treated by a mixture of independent and constrained refinement
Δρ_max_ = 1.10 e Å^−3^
Δρ_min_ = −0.70 e Å^−3^

### 

Data collection: *APEX2* (Bruker, 2004 ▶); cell refinement: *SAINT* (Bruker, 2004 ▶); data reduction: *SAINT*; program(s) used to solve structure: *SHELXS97* (Sheldrick, 2008 ▶); program(s) used to refine structure: *SHELXL97* (Sheldrick, 2008 ▶); molecular graphics: *X-SEED* (Barbour, 2001 ▶); software used to prepare material for publication: *publCIF* (Westrip, 2009 ▶).

## Supplementary Material

Crystal structure: contains datablocks I, global. DOI: 10.1107/S1600536809052635/ci2985sup1.cif

Structure factors: contains datablocks I. DOI: 10.1107/S1600536809052635/ci2985Isup2.hkl

Additional supplementary materials:  crystallographic information; 3D view; checkCIF report

## Figures and Tables

**Table 1:** Selected bond lengths (Å)

Zn1—N1	2.033 (3)
Zn1—N3^i^	2.024 (3)
Zn1—Cl1	2.248 (1)
Zn1—Cl2	2.247 (1)

Symmetry code: (i) 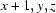.

**Table 2:** Hydrogen-bond geometry (Å, °)

*D*—H⋯*A*	*D*—H	H⋯*A*	*D*⋯*A*	*D*—H⋯*A*
N2—H2⋯N5	0.88 (1)	1.92 (1)	2.787 (5)	169 (4)
N4—H4⋯N8^ii^	0.88 (1)	1.90 (1)	2.773 (5)	175 (4)
N6—H6⋯Cl1^iii^	0.88 (1)	2.37 (2)	3.224 (4)	167 (5)
N7—H7⋯Cl2^iv^	0.88 (1)	2.35 (1)	3.230 (4)	178 (4)

Symmetry codes: (ii) 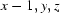; (iii) 
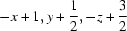
; (iv) 
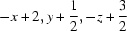
.

**Fig. 1.**
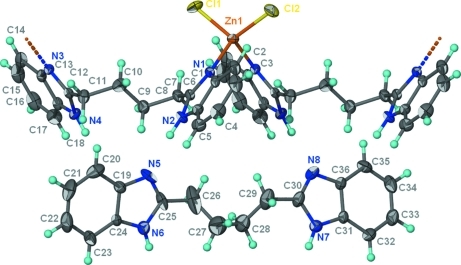
Displacement ellipsoid plot (Barbour, 2001) of a portion of the polymeric structure of ZnCl2(C18H18N4).C18H18N4 at the 50% probability level. H atoms are drawn as sphere of arbitrary radius. Only the major disorder component of the butyl chain is shown.

## supplementary crystallographic information

### Experimental

1,4-Bis(2-benzimidazolyl)butane was synthesized by using a literature method (van Albada *et al.*, 1995). To a solution of zinc chloride hexahydrate (0.25 g, 1 mmol) in ethanol (3 ml) was added an aqueous solution (4 ml) of the ligand (0.27 g, 1 mmol). The reactants were sealed in a 15-ml Teflon-lined, stainless-steel Parr bomb. The bomb was heated at 413 K for 3 d. The cool solution yielded red block single crystals in *ca* 30% yield.

### Refinement

Carbon-bound hydrogen atoms were generated geometrically and were constrained to ride on their parent atoms [C–H = 0.95–0.99 Å and *U*_iso_(H) = 1.2*U*_eq_(C)]. The nitrogen-bound ones were located in a difference Fourier map, and were refined with a N-H distance restraint of 0.88 (1) Å; their temperature factors were similarly tied.

The butyl chain of the uncoordinated ligand is disordered over three positions with occupancies of 0.511 (4), 0.289 (5) and 0.200 (5). The 1,2-related distances were restrained to 1.50 (1) Å and the 1,3-related ones to 2.35 (1) Å. The final difference Fourier map had a peak in the vicinity of the disordered atoms.

### Crystal data

**Table:**

[ZnCl_2_(C_18_H_18_N_4_)]·C_18_H_18_N_4_	*F*(000) = 1488
*M**_r_* = 717.00	*D*_x_ = 1.373 Mg m^−^^3^
Monoclinic, *P*2_1_/*c*	Mo *K*α radiation, λ = 0.71073 Å
Hall symbol: -P 2ybc	Cell parameters from 3392 reflections
*a* = 8.5321 (5) Å	θ = 2.4–25.2°
*b* = 24.119 (2) Å	µ = 0.90 mm^−^^1^
*c* = 16.880 (1) Å	*T* = 173 K
β = 92.999 (1)°	Block, red
*V* = 3468.9 (4) Å^3^	0.45 × 0.30 × 0.20 mm
*Z* = 4	

### Data collection

**Table:**

Bruker APEXII area-detector diffractometer	7549 independent reflections
Radiation source: fine-focus sealed tube	3855 reflections with *I* > 2σ(*I*)
graphite	*R*_int_ = 0.052
φ and ω scans	θ_max_ = 27.0°, θ_min_ = 1.5°
Absorption correction: multi-scan (*SADABS*; Sheldrick, 1996)	*h* = −10→10
*T*_min_ = 0.687, *T*_max_ = 0.840	*k* = −30→18
17738 measured reflections	*l* = −19→21

### Refinement

**Table:**

Refinement on *F*^2^	Primary atom site location: structure-invariant direct methods
Least-squares matrix: full	Secondary atom site location: difference Fourier map
*R*[*F*^2^ > 2σ(*F*^2^)] = 0.062	Hydrogen site location: inferred from neighbouring sites
*wR*(*F*^2^) = 0.192	H atoms treated by a mixture of independent and constrained refinement
*S* = 1.02	*w* = 1/[σ^2^(*F*_o_^2^) + (0.0962*P*)^2^] where *P* = (*F*_o_^2^ + 2*F*_c_^2^)/3
7549 reflections	(Δ/σ)_max_ = 0.001
448 parameters	Δρ_max_ = 1.10 e Å^−^^3^
32 restraints	Δρ_min_ = −0.69 e Å^−^^3^

### Fractional atomic coordinates and isotropic or equivalent isotropic displacement parameters (Å^2^)

**Table:**

	*x*	*y*	*z*	*U*_iso_*/*U*_eq_	Occ. (<1)
Zn1	0.76884 (6)	0.443756 (19)	0.75855 (3)	0.03034 (19)	
Cl1	0.58239 (17)	0.39129 (5)	0.81075 (9)	0.0579 (4)	
Cl2	0.95928 (16)	0.39314 (5)	0.70655 (9)	0.0518 (4)	
N1	0.6603 (4)	0.49713 (13)	0.6803 (2)	0.0239 (8)	
N2	0.5260 (4)	0.57190 (14)	0.6425 (2)	0.0293 (9)	
H2	0.468 (4)	0.6019 (11)	0.637 (3)	0.035*	
N3	−0.1295 (4)	0.49956 (13)	0.8346 (2)	0.0243 (8)	
N4	−0.0076 (4)	0.57767 (15)	0.8695 (2)	0.0297 (9)	
H4	0.042 (5)	0.6091 (10)	0.864 (3)	0.036*	
N5	0.3642 (5)	0.67223 (16)	0.6438 (2)	0.0434 (11)	
N6	0.3359 (5)	0.76206 (16)	0.6609 (3)	0.0419 (11)	
H6	0.360 (6)	0.7956 (9)	0.677 (3)	0.050*	
N7	1.1593 (5)	0.76918 (15)	0.8373 (2)	0.0405 (10)	
H7	1.125 (5)	0.8026 (9)	0.825 (3)	0.049*	
N8	1.1417 (5)	0.67940 (16)	0.8606 (2)	0.0388 (10)	
C1	0.6918 (5)	0.50889 (16)	0.6017 (2)	0.0247 (10)	
C2	0.7893 (5)	0.48250 (19)	0.5501 (3)	0.0360 (11)	
H2a	0.8472	0.4502	0.5655	0.043*	
C3	0.7981 (6)	0.5056 (2)	0.4750 (3)	0.0437 (13)	
H3	0.8641	0.4887	0.4383	0.052*	
C4	0.7138 (6)	0.5523 (2)	0.4520 (3)	0.0430 (13)	
H4A	0.7230	0.5667	0.4001	0.052*	
C5	0.6168 (6)	0.57815 (19)	0.5029 (3)	0.0381 (12)	
H5	0.5585	0.6102	0.4872	0.046*	
C6	0.6070 (5)	0.55583 (17)	0.5777 (3)	0.0284 (10)	
C7	0.5620 (5)	0.53660 (16)	0.7022 (3)	0.0256 (10)	
C8	0.4998 (5)	0.54272 (18)	0.7826 (3)	0.0315 (11)	
H8A	0.5665	0.5696	0.8132	0.038*	
H8B	0.5093	0.5066	0.8102	0.038*	
C9	0.3315 (5)	0.56169 (18)	0.7834 (3)	0.0339 (11)	
H9A	0.3029	0.5665	0.8390	0.041*	
H9B	0.3214	0.5982	0.7567	0.041*	
C10	0.2204 (5)	0.5219 (2)	0.7431 (3)	0.0371 (11)	
H10A	0.2259	0.4864	0.7724	0.044*	
H10B	0.2556	0.5147	0.6891	0.044*	
C11	0.0455 (5)	0.54116 (19)	0.7360 (3)	0.0330 (11)	
H11A	0.0410	0.5796	0.7154	0.040*	
H11B	−0.0131	0.5172	0.6971	0.040*	
C12	−0.0323 (5)	0.53918 (16)	0.8124 (2)	0.0243 (10)	
C13	−0.1716 (5)	0.51441 (17)	0.9110 (3)	0.0272 (10)	
C14	−0.2741 (5)	0.4896 (2)	0.9620 (3)	0.0396 (12)	
H14	−0.3266	0.4559	0.9486	0.047*	
C15	−0.2956 (6)	0.5164 (2)	1.0328 (3)	0.0509 (15)	
H15	−0.3652	0.5005	1.0686	0.061*	
C16	−0.2204 (7)	0.5652 (2)	1.0538 (3)	0.0521 (15)	
H16	−0.2395	0.5822	1.1032	0.063*	
C17	−0.1178 (6)	0.5895 (2)	1.0040 (3)	0.0445 (13)	
H17	−0.0646	0.6229	1.0182	0.053*	
C18	−0.0950 (5)	0.56348 (17)	0.9328 (3)	0.0287 (10)	
C19	0.2264 (5)	0.68930 (18)	0.6044 (3)	0.0313 (11)	
C20	0.1127 (7)	0.6592 (2)	0.5586 (3)	0.0571 (16)	
H20	0.1210	0.6203	0.5509	0.069*	
C21	−0.0121 (7)	0.6897 (3)	0.5254 (4)	0.0686 (19)	
H21	−0.0902	0.6711	0.4932	0.082*	
C22	−0.0268 (6)	0.7454 (3)	0.5375 (4)	0.0639 (18)	
H22	−0.1158	0.7640	0.5141	0.077*	
C23	0.0817 (6)	0.7754 (2)	0.5818 (3)	0.0511 (15)	
H23	0.0705	0.8142	0.5901	0.061*	
C24	0.2081 (5)	0.74616 (18)	0.6138 (3)	0.0315 (11)	
C25	0.4246 (6)	0.7171 (2)	0.6767 (3)	0.0455 (14)	
C26	0.5745 (7)	0.7182 (3)	0.7284 (4)	0.093 (3)	
H26A	0.5445	0.7204	0.7842	0.112*	0.511 (4)
H26B	0.6274	0.6821	0.7221	0.112*	0.511 (4)
H26C	0.6509	0.7328	0.6915	0.112*	0.289 (5)
H26D	0.5552	0.7495	0.7646	0.112*	0.289 (5)
H26E	0.5844	0.7544	0.7561	0.112*	0.200 (5)
H26F	0.5717	0.6887	0.7691	0.112*	0.200 (5)
C27	0.6908 (8)	0.7616 (3)	0.7171 (5)	0.086 (4)	0.511 (4)
H27A	0.6385	0.7981	0.7125	0.103*	0.511 (4)
H27B	0.7453	0.7546	0.6677	0.103*	0.511 (4)
C28	0.8063 (8)	0.7613 (3)	0.7867 (5)	0.065 (3)	0.511 (4)
H28A	0.8554	0.7983	0.7933	0.078*	0.511 (4)
H28B	0.7525	0.7523	0.8357	0.078*	0.511 (4)
C27'	0.6698 (13)	0.6826 (6)	0.7778 (11)	0.086 (4)	0.289 (5)
H27C	0.7209	0.6548	0.7447	0.103*	0.289 (5)
H27D	0.6033	0.6626	0.8148	0.103*	0.289 (5)
C28'	0.7909 (11)	0.7148 (8)	0.8236 (7)	0.065 (3)	0.289 (5)
H28C	0.8217	0.6955	0.8738	0.078*	0.289 (5)
H28D	0.7506	0.7520	0.8363	0.078*	0.289 (5)
C27"	0.7118 (12)	0.7096 (11)	0.6788 (7)	0.086 (4)	0.200 (5)
H27E	0.6809	0.6876	0.6310	0.103*	0.200 (5)
H27F	0.7540	0.7457	0.6618	0.103*	0.200 (5)
C28"	0.8301 (14)	0.6796 (6)	0.7288 (14)	0.065 (3)	0.200 (5)
H28E	0.7775	0.6548	0.7659	0.078*	0.200 (5)
H28F	0.8959	0.6566	0.6951	0.078*	0.200 (5)
C29	0.9271 (6)	0.7194 (3)	0.7729 (4)	0.0680 (19)	
H29A	0.9552	0.7223	0.7169	0.082*	0.511 (4)
H29B	0.8799	0.6823	0.7798	0.082*	0.511 (4)
H29C	0.9167	0.7532	0.7397	0.082*	0.289 (5)
H29D	0.9303	0.6869	0.7372	0.082*	0.289 (5)
H29E	0.8505	0.7369	0.8071	0.082*	0.200 (5)
H29F	0.9465	0.7475	0.7319	0.082*	0.200 (5)
C30	1.0769 (6)	0.7222 (2)	0.8247 (3)	0.0421 (13)	
C31	1.2901 (5)	0.75633 (18)	0.8853 (3)	0.0320 (11)	
C32	1.4133 (6)	0.7881 (2)	0.9160 (3)	0.0442 (13)	
H32	1.4210	0.8266	0.9048	0.053*	
C33	1.5239 (6)	0.7611 (2)	0.9633 (3)	0.0548 (16)	
H33	1.6099	0.7816	0.9863	0.066*	
C34	1.5145 (6)	0.7049 (2)	0.9788 (3)	0.0538 (16)	
H34	1.5945	0.6878	1.0115	0.065*	
C35	1.3913 (6)	0.6729 (2)	0.9477 (3)	0.0463 (14)	
H35	1.3850	0.6344	0.9589	0.056*	
C36	1.2775 (5)	0.69929 (18)	0.8997 (3)	0.0328 (11)	

### Atomic displacement parameters (Å^2^)

**Table:**

	*U*^11^	*U*^22^	*U*^33^	*U*^12^	*U*^13^	*U*^23^
Zn1	0.0306 (3)	0.0192 (3)	0.0406 (4)	0.0004 (2)	−0.0038 (2)	−0.0001 (2)
Cl1	0.0530 (9)	0.0321 (7)	0.0887 (11)	−0.0170 (6)	0.0056 (8)	0.0139 (7)
Cl2	0.0455 (8)	0.0338 (7)	0.0754 (10)	0.0136 (6)	−0.0030 (7)	−0.0160 (6)
N1	0.0207 (18)	0.0230 (18)	0.028 (2)	0.0031 (14)	−0.0005 (16)	−0.0037 (15)
N2	0.027 (2)	0.029 (2)	0.032 (2)	0.0114 (16)	0.0011 (18)	0.0021 (17)
N3	0.0224 (19)	0.0229 (18)	0.027 (2)	−0.0016 (15)	−0.0021 (16)	0.0008 (15)
N4	0.028 (2)	0.027 (2)	0.034 (2)	−0.0068 (16)	0.0033 (18)	−0.0030 (17)
N5	0.054 (3)	0.040 (2)	0.036 (2)	0.029 (2)	0.000 (2)	0.0014 (19)
N6	0.032 (2)	0.037 (2)	0.056 (3)	0.0006 (19)	−0.007 (2)	−0.015 (2)
N7	0.036 (2)	0.033 (2)	0.051 (3)	−0.0021 (19)	−0.010 (2)	0.007 (2)
N8	0.040 (2)	0.038 (2)	0.038 (2)	−0.0135 (19)	0.001 (2)	−0.0025 (18)
C1	0.019 (2)	0.029 (2)	0.026 (2)	−0.0010 (17)	−0.0019 (19)	−0.0047 (18)
C2	0.030 (3)	0.044 (3)	0.035 (3)	0.008 (2)	0.001 (2)	−0.010 (2)
C3	0.037 (3)	0.063 (3)	0.032 (3)	0.008 (3)	0.006 (2)	−0.012 (3)
C4	0.050 (3)	0.053 (3)	0.027 (3)	−0.007 (3)	0.006 (2)	−0.003 (2)
C5	0.044 (3)	0.036 (3)	0.034 (3)	0.001 (2)	−0.004 (2)	0.004 (2)
C6	0.027 (2)	0.027 (2)	0.031 (3)	−0.0042 (19)	0.002 (2)	−0.002 (2)
C7	0.021 (2)	0.025 (2)	0.031 (3)	0.0023 (18)	−0.001 (2)	−0.0044 (18)
C8	0.027 (2)	0.033 (3)	0.035 (3)	0.0068 (19)	0.008 (2)	−0.0011 (19)
C9	0.028 (2)	0.040 (3)	0.035 (3)	0.003 (2)	0.009 (2)	0.000 (2)
C10	0.046 (3)	0.038 (3)	0.029 (3)	−0.006 (2)	0.016 (2)	−0.010 (2)
C11	0.029 (3)	0.042 (3)	0.029 (3)	−0.002 (2)	0.003 (2)	−0.002 (2)
C12	0.020 (2)	0.024 (2)	0.028 (2)	0.0036 (17)	−0.0012 (19)	0.0013 (18)
C13	0.020 (2)	0.034 (2)	0.027 (2)	0.0016 (19)	−0.001 (2)	0.0052 (19)
C14	0.031 (3)	0.053 (3)	0.035 (3)	0.001 (2)	0.002 (2)	0.017 (2)
C15	0.038 (3)	0.083 (4)	0.032 (3)	0.008 (3)	0.010 (3)	0.019 (3)
C16	0.048 (3)	0.076 (4)	0.033 (3)	0.015 (3)	0.004 (3)	−0.002 (3)
C17	0.050 (3)	0.048 (3)	0.035 (3)	0.005 (3)	0.002 (3)	−0.008 (2)
C18	0.022 (2)	0.037 (3)	0.027 (3)	0.0029 (19)	−0.001 (2)	0.002 (2)
C19	0.032 (3)	0.032 (2)	0.030 (3)	0.005 (2)	0.001 (2)	0.004 (2)
C20	0.068 (4)	0.042 (3)	0.061 (4)	−0.017 (3)	0.003 (3)	−0.012 (3)
C21	0.041 (4)	0.095 (5)	0.068 (5)	−0.023 (4)	−0.013 (3)	−0.011 (4)
C22	0.033 (3)	0.092 (5)	0.066 (4)	0.002 (3)	−0.013 (3)	0.014 (4)
C23	0.033 (3)	0.052 (3)	0.068 (4)	0.010 (2)	−0.006 (3)	0.017 (3)
C24	0.022 (2)	0.029 (2)	0.043 (3)	0.0016 (18)	−0.006 (2)	0.003 (2)
C25	0.030 (3)	0.069 (4)	0.037 (3)	0.022 (3)	−0.007 (2)	−0.008 (3)
C26	0.047 (4)	0.162 (7)	0.070 (5)	0.035 (5)	−0.011 (4)	−0.052 (5)
C27	0.072 (6)	0.078 (6)	0.106 (7)	−0.007 (6)	−0.017 (6)	0.014 (6)
C28	0.050 (5)	0.061 (5)	0.082 (6)	−0.006 (5)	−0.014 (5)	−0.012 (5)
C27'	0.072 (6)	0.078 (6)	0.106 (7)	−0.007 (6)	−0.017 (6)	0.014 (6)
C28'	0.050 (5)	0.061 (5)	0.082 (6)	−0.006 (5)	−0.014 (5)	−0.012 (5)
C27"	0.072 (6)	0.078 (6)	0.106 (7)	−0.007 (6)	−0.017 (6)	0.014 (6)
C28"	0.050 (5)	0.061 (5)	0.082 (6)	−0.006 (5)	−0.014 (5)	−0.012 (5)
C29	0.045 (4)	0.097 (5)	0.061 (4)	−0.019 (3)	−0.011 (3)	0.030 (3)
C30	0.032 (3)	0.053 (3)	0.040 (3)	−0.012 (2)	−0.007 (2)	0.007 (2)
C31	0.021 (2)	0.034 (3)	0.040 (3)	−0.0011 (19)	−0.004 (2)	−0.006 (2)
C32	0.039 (3)	0.034 (3)	0.059 (4)	−0.006 (2)	−0.005 (3)	−0.006 (2)
C33	0.034 (3)	0.056 (4)	0.073 (4)	−0.006 (3)	−0.012 (3)	−0.019 (3)
C34	0.037 (3)	0.065 (4)	0.058 (4)	0.013 (3)	−0.015 (3)	−0.005 (3)
C35	0.049 (3)	0.034 (3)	0.056 (4)	0.010 (2)	−0.002 (3)	0.005 (2)
C36	0.026 (3)	0.033 (3)	0.039 (3)	−0.003 (2)	0.000 (2)	−0.002 (2)

### Geometric parameters (Å, °)

**Table:**

Zn1—N1	2.033 (3)	C17—H17	0.95
Zn1—N3^i^	2.024 (3)	C19—C24	1.390 (6)
Zn1—Cl1	2.248 (1)	C19—C20	1.409 (7)
Zn1—Cl2	2.247 (1)	C20—C21	1.387 (8)
N1—C7	1.334 (5)	C20—H20	0.95
N1—C1	1.395 (5)	C21—C22	1.365 (8)
N2—C7	1.342 (5)	C21—H21	0.95
N2—C6	1.381 (6)	C22—C23	1.368 (8)
N2—H2	0.876 (10)	C22—H22	0.95
N3—C12	1.332 (5)	C23—C24	1.375 (6)
N3—C13	1.402 (5)	C23—H23	0.95
N3—Zn1^ii^	2.024 (3)	C25—C26	1.511 (7)
N4—C12	1.347 (5)	C26—C27'	1.423 (9)
N4—C18	1.378 (6)	C26—C27	1.460 (7)
N4—H4	0.878 (10)	C26—C27"	1.490 (10)
N5—C25	1.310 (6)	C26—H26A	0.99
N5—C19	1.383 (6)	C26—H26B	0.99
N6—C25	1.341 (6)	C26—H26C	0.99
N6—C24	1.370 (5)	C26—H26D	0.99
N6—H6	0.875 (10)	C26—H26E	0.99
N7—C30	1.345 (6)	C26—H26F	0.99
N7—C31	1.379 (6)	C27—C28	1.4936
N7—H7	0.879 (10)	C27—H27A	0.99
N8—C30	1.305 (6)	C27—H27B	0.99
N8—C36	1.388 (6)	C28—C29	1.471 (7)
C1—C2	1.390 (6)	C28—H28A	0.99
C1—C6	1.392 (5)	C28—H28B	0.99
C2—C3	1.391 (7)	C27'—C28'	1.477 (9)
C2—H2a	0.95	C27'—H27C	0.99
C3—C4	1.381 (7)	C27'—H27D	0.99
C3—H3	0.95	C28'—C29	1.483 (9)
C4—C5	1.375 (7)	C28'—H28C	0.99
C4—H4A	0.95	C28'—H28D	0.99
C5—C6	1.378 (6)	C27"—C28"	1.471 (10)
C5—H5	0.95	C27"—H27E	0.99
C7—C8	1.490 (6)	C27"—H27F	0.99
C8—C9	1.508 (6)	C28"—C29	1.448 (10)
C8—H8A	0.99	C28"—H28E	0.99
C8—H8B	0.99	C28"—H28F	0.99
C9—C10	1.488 (6)	C29—C30	1.512 (7)
C9—H9A	0.99	C29—H29A	0.99
C9—H9B	0.99	C29—H29B	0.99
C10—C11	1.561 (6)	C29—H29C	0.99
C10—H10A	0.99	C29—H29D	0.99
C10—H10B	0.99	C29—H29E	0.99
C11—C12	1.482 (6)	C29—H29F	0.99
C11—H11A	0.99	C31—C32	1.380 (6)
C11—H11B	0.99	C31—C36	1.402 (6)
C13—C18	1.392 (6)	C32—C33	1.369 (7)
C13—C14	1.394 (6)	C32—H32	0.95
C14—C15	1.378 (7)	C33—C34	1.384 (7)
C14—H14	0.95	C33—H33	0.95
C15—C16	1.379 (7)	C34—C35	1.384 (7)
C15—H15	0.95	C34—H34	0.95
C16—C17	1.376 (7)	C35—C36	1.386 (6)
C16—H16	0.95	C35—H35	0.95
C17—C18	1.379 (6)		
			
N3^i^—Zn1—N1	99.03 (13)	C23—C22—H22	118.7
N3^i^—Zn1—Cl2	108.33 (10)	C22—C23—C24	115.9 (5)
N1—Zn1—Cl2	113.83 (10)	C22—C23—H23	122.0
N3^i^—Zn1—Cl1	114.47 (11)	C24—C23—H23	122.0
N1—Zn1—Cl1	107.68 (11)	N6—C24—C23	131.9 (4)
Cl2—Zn1—Cl1	112.84 (6)	N6—C24—C19	104.6 (4)
C7—N1—C1	105.9 (3)	C23—C24—C19	123.5 (4)
C7—N1—Zn1	122.9 (3)	N5—C25—N6	112.3 (4)
C1—N1—Zn1	130.0 (3)	N5—C25—C26	124.2 (5)
C7—N2—C6	108.2 (3)	N6—C25—C26	123.5 (5)
C7—N2—H2	135 (3)	C27—C26—C25	119.8 (6)
C6—N2—H2	117 (3)	C27"—C26—C25	109.8 (6)
C12—N3—C13	105.6 (3)	C27—C26—H26A	107.4
C12—N3—Zn1^ii^	123.4 (3)	C25—C26—H26A	107.4
C13—N3—Zn1^ii^	129.1 (3)	C27—C26—H26B	107.4
C12—N4—C18	108.2 (4)	C25—C26—H26B	107.4
C12—N4—H4	125 (3)	H26A—C26—H26B	106.9
C18—N4—H4	126 (3)	C27'—C26—H26C	102.0
C25—N5—C19	105.3 (4)	C25—C26—H26C	102.0
C25—N6—C24	108.2 (4)	C27'—C26—H26D	102.0
C25—N6—H6	124 (3)	C25—C26—H26D	102.0
C24—N6—H6	127 (3)	H26C—C26—H26D	104.8
C30—N7—C31	107.7 (4)	C27'—C26—H26E	102.9
C30—N7—H7	125 (3)	C27"—C26—H26E	109.7
C31—N7—H7	127 (3)	C25—C26—H26E	109.7
C30—N8—C36	105.7 (4)	C27"—C26—H26F	109.7
C2—C1—C6	120.5 (4)	C25—C26—H26F	109.7
C2—C1—N1	130.8 (4)	H26E—C26—H26F	108.2
C6—C1—N1	108.7 (4)	C26—C27—C28	108.6 (4)
C1—C2—C3	116.8 (4)	C26—C27—H27A	110.0
C1—C2—H2a	121.6	C28—C27—H27A	110.0
C3—C2—H2a	121.6	C26—C27—H27B	110.0
C4—C3—C2	122.1 (5)	C28—C27—H27B	110.0
C4—C3—H3	119.0	H27A—C27—H27B	108.4
C2—C3—H3	119.0	C29—C28—C27	108.5 (4)
C5—C4—C3	121.1 (5)	C29—C28—H28A	110.0
C5—C4—H4A	119.5	C27—C28—H28A	110.0
C3—C4—H4A	119.5	C29—C28—H28B	110.0
C4—C5—C6	117.6 (4)	C27—C28—H28B	110.0
C4—C5—H5	121.2	H28A—C28—H28B	108.4
C6—C5—H5	121.2	C26—C27'—C28'	110.6 (9)
C5—C6—N2	132.3 (4)	C26—C27'—H27C	109.5
C5—C6—C1	122.0 (4)	C28'—C27'—H27C	109.5
N2—C6—C1	105.7 (4)	C26—C27'—H27D	109.5
N1—C7—N2	111.5 (4)	C28'—C27'—H27D	109.5
N1—C7—C8	125.6 (4)	H27C—C27'—H27D	108.1
N2—C7—C8	122.8 (4)	C27'—C28'—C29	106.6 (9)
C7—C8—C9	115.0 (4)	C27'—C28'—H28C	110.4
C7—C8—H8A	108.5	C29—C28'—H28C	110.4
C9—C8—H8A	108.5	C27'—C28'—H28D	110.4
C7—C8—H8B	108.5	C29—C28'—H28D	110.4
C9—C8—H8B	108.5	H28C—C28'—H28D	108.6
H8A—C8—H8B	107.5	C28"—C27"—C26	106.4 (9)
C10—C9—C8	112.6 (4)	C28"—C27"—H27E	110.5
C10—C9—H9A	109.1	C26—C27"—H27E	110.5
C8—C9—H9A	109.1	C28"—C27"—H27F	110.5
C10—C9—H9B	109.1	C26—C27"—H27F	110.5
C8—C9—H9B	109.1	H27E—C27"—H27F	108.6
H9A—C9—H9B	107.8	C29—C28"—C27"	109.0 (10)
C9—C10—C11	115.3 (4)	C29—C28"—H28E	109.9
C9—C10—H10A	108.5	C27"—C28"—H28E	109.9
C11—C10—H10A	108.5	C29—C28"—H28F	109.9
C9—C10—H10B	108.5	C27"—C28"—H28F	109.9
C11—C10—H10B	108.5	H28E—C28"—H28F	108.3
H10A—C10—H10B	107.5	C28—C29—C30	117.1 (5)
C12—C11—C10	113.2 (4)	C28'—C29—C30	109.5 (6)
C12—C11—H11A	108.9	C28—C29—H29A	108.0
C10—C11—H11A	108.9	C30—C29—H29A	108.0
C12—C11—H11B	108.9	C28—C29—H29B	108.0
C10—C11—H11B	108.9	C30—C29—H29B	108.0
H11A—C11—H11B	107.8	H29A—C29—H29B	107.3
N3—C12—N4	111.7 (4)	C28"—C29—H29C	102.8
N3—C12—C11	125.6 (4)	C28'—C29—H29C	109.8
N4—C12—C11	122.7 (4)	C30—C29—H29C	109.8
C18—C13—C14	120.1 (4)	C28'—C29—H29D	109.8
C18—C13—N3	108.7 (4)	C30—C29—H29D	109.8
C14—C13—N3	131.1 (4)	H29C—C29—H29D	108.2
C15—C14—C13	116.8 (5)	C28"—C29—H29E	101.9
C15—C14—H14	121.6	C30—C29—H29E	101.9
C13—C14—H14	121.6	C28"—C29—H29F	101.9
C14—C15—C16	122.8 (5)	C30—C29—H29F	101.9
C14—C15—H15	118.6	H29E—C29—H29F	104.7
C16—C15—H15	118.6	N8—C30—N7	112.8 (4)
C17—C16—C15	120.5 (5)	N8—C30—C29	123.9 (5)
C17—C16—H16	119.7	N7—C30—C29	123.3 (5)
C15—C16—H16	119.7	N7—C31—C32	132.3 (4)
C16—C17—C18	117.6 (5)	N7—C31—C36	104.8 (4)
C16—C17—H17	121.2	C32—C31—C36	122.9 (4)
C18—C17—H17	121.2	C33—C32—C31	116.3 (5)
N4—C18—C17	132.1 (4)	C33—C32—H32	121.8
N4—C18—C13	105.8 (4)	C31—C32—H32	121.8
C17—C18—C13	122.1 (4)	C32—C33—C34	122.0 (5)
N5—C19—C24	109.7 (4)	C32—C33—H33	119.0
N5—C19—C20	130.8 (5)	C34—C33—H33	119.0
C24—C19—C20	119.5 (4)	C33—C34—C35	121.7 (5)
C21—C20—C19	116.1 (5)	C33—C34—H34	119.2
C21—C20—H20	121.9	C35—C34—H34	119.2
C19—C20—H20	121.9	C34—C35—C36	117.4 (5)
C22—C21—C20	122.4 (5)	C34—C35—H35	121.3
C22—C21—H21	118.8	C36—C35—H35	121.3
C20—C21—H21	118.8	C35—C36—N8	131.4 (4)
C21—C22—C23	122.6 (5)	C35—C36—C31	119.6 (4)
C21—C22—H22	118.7	N8—C36—C31	109.1 (4)
			
N3^i^—Zn1—N1—C7	−54.6 (3)	C25—N5—C19—C20	180.0 (5)
Cl2—Zn1—N1—C7	−169.3 (3)	N5—C19—C20—C21	−178.7 (5)
Cl1—Zn1—N1—C7	64.8 (3)	C24—C19—C20—C21	0.2 (8)
N3^i^—Zn1—N1—C1	110.9 (3)	C19—C20—C21—C22	−1.4 (9)
Cl2—Zn1—N1—C1	−3.9 (4)	C20—C21—C22—C23	1.2 (10)
Cl1—Zn1—N1—C1	−129.7 (3)	C21—C22—C23—C24	0.2 (9)
C7—N1—C1—C2	178.1 (4)	C25—N6—C24—C23	179.2 (6)
Zn1—N1—C1—C2	10.8 (6)	C25—N6—C24—C19	1.0 (5)
C7—N1—C1—C6	−1.0 (4)	C22—C23—C24—N6	−179.4 (6)
Zn1—N1—C1—C6	−168.3 (3)	C22—C23—C24—C19	−1.5 (8)
C6—C1—C2—C3	0.6 (6)	N5—C19—C24—N6	−1.2 (5)
N1—C1—C2—C3	−178.4 (4)	C20—C19—C24—N6	179.7 (5)
C1—C2—C3—C4	−0.4 (7)	N5—C19—C24—C23	−179.6 (5)
C2—C3—C4—C5	0.0 (8)	C20—C19—C24—C23	1.3 (8)
C3—C4—C5—C6	0.1 (7)	C19—N5—C25—N6	−0.4 (6)
C4—C5—C6—N2	178.1 (5)	C19—N5—C25—C26	178.0 (5)
C4—C5—C6—C1	0.1 (7)	C24—N6—C25—N5	−0.4 (6)
C7—N2—C6—C5	−177.7 (5)	C24—N6—C25—C26	−178.8 (5)
C7—N2—C6—C1	0.5 (5)	N5—C25—C26—C27'	−24.1 (18)
C2—C1—C6—C5	−0.5 (6)	N6—C25—C26—C27'	154.1 (15)
N1—C1—C6—C5	178.7 (4)	N5—C25—C26—C27	135.4 (7)
C2—C1—C6—N2	−178.9 (4)	N6—C25—C26—C27	−46.4 (9)
N1—C1—C6—N2	0.3 (4)	N5—C25—C26—C27"	71.9 (14)
C1—N1—C7—N2	1.4 (5)	N6—C25—C26—C27"	−109.8 (13)
Zn1—N1—C7—N2	169.8 (3)	C25—C26—C27—C28	166.7 (4)
C1—N1—C7—C8	−177.5 (4)	C26—C27—C28—C29	84.5 (6)
Zn1—N1—C7—C8	−9.1 (6)	C25—C26—C27'—C28'	−169.7 (9)
C6—N2—C7—N1	−1.2 (5)	C26—C27'—C28'—C29	−87.3 (14)
C6—N2—C7—C8	177.7 (4)	C25—C26—C27"—C28"	−147.5 (10)
N1—C7—C8—C9	−142.7 (4)	C26—C27"—C28"—C29	−88.2 (15)
N2—C7—C8—C9	38.5 (6)	C27"—C28"—C29—C28	35.5 (15)
C7—C8—C9—C10	61.9 (5)	C27"—C28"—C29—C30	−169.5 (9)
C8—C9—C10—C11	−175.5 (4)	C27—C28—C29—C30	164.5 (4)
C9—C10—C11—C12	−73.1 (5)	C27'—C28'—C29—C28	100.9 (10)
C13—N3—C12—N4	1.0 (4)	C27'—C28'—C29—C30	−149.4 (8)
Zn1^ii^—N3—C12—N4	166.7 (3)	C36—N8—C30—N7	−0.2 (6)
C13—N3—C12—C11	179.0 (4)	C36—N8—C30—C29	178.4 (5)
Zn1^ii^—N3—C12—C11	−15.2 (6)	C31—N7—C30—N8	0.2 (6)
C18—N4—C12—N3	−1.0 (5)	C31—N7—C30—C29	−178.4 (5)
C18—N4—C12—C11	−179.2 (4)	C28"—C29—C30—N8	−20 (2)
C10—C11—C12—N3	−101.0 (5)	C28—C29—C30—N8	132.5 (6)
C10—C11—C12—N4	76.9 (5)	C28'—C29—C30—N8	76.2 (10)
C12—N3—C13—C18	−0.6 (4)	C28"—C29—C30—N7	158.9 (17)
Zn1^ii^—N3—C13—C18	−165.2 (3)	C28—C29—C30—N7	−49.0 (8)
C12—N3—C13—C14	177.4 (4)	C28'—C29—C30—N7	−105.3 (9)
Zn1^ii^—N3—C13—C14	12.8 (6)	C30—N7—C31—C32	179.6 (5)
C18—C13—C14—C15	0.8 (6)	C30—N7—C31—C36	−0.2 (5)
N3—C13—C14—C15	−177.0 (4)	N7—C31—C32—C33	179.1 (5)
C13—C14—C15—C16	−0.3 (7)	C36—C31—C32—C33	−1.1 (8)
C14—C15—C16—C17	−0.4 (8)	C31—C32—C33—C34	1.0 (9)
C15—C16—C17—C18	0.6 (7)	C32—C33—C34—C35	−0.7 (9)
C12—N4—C18—C17	−176.8 (5)	C33—C34—C35—C36	0.5 (9)
C12—N4—C18—C13	0.6 (5)	C34—C35—C36—N8	−179.7 (5)
C16—C17—C18—N4	176.9 (5)	C34—C35—C36—C31	−0.7 (8)
C16—C17—C18—C13	−0.1 (7)	C30—N8—C36—C35	179.1 (5)
C14—C13—C18—N4	−178.3 (4)	C30—N8—C36—C31	0.1 (5)
N3—C13—C18—N4	0.0 (4)	N7—C31—C36—C35	−179.1 (5)
C14—C13—C18—C17	−0.6 (6)	C32—C31—C36—C35	1.0 (8)
N3—C13—C18—C17	177.7 (4)	N7—C31—C36—N8	0.1 (5)
C25—N5—C19—C24	1.0 (6)	C32—C31—C36—N8	−179.8 (5)

Symmetry codes: (i) *x*+1, *y*, *z*; (ii) *x*−1, *y*, *z*.

### Hydrogen-bond geometry (Å, °)

**Table:**

*D*—H···*A*	*D*—H	H···*A*	*D*···*A*	*D*—H···*A*
N2—H2···N5	0.88 (1)	1.92 (1)	2.787 (5)	169 (4)
N4—H4···N8^ii^	0.88 (1)	1.90 (1)	2.773 (5)	175 (4)
N6—H6···Cl1^iii^	0.88 (1)	2.37 (2)	3.224 (4)	167 (5)
N7—H7···Cl2^iv^	0.88 (1)	2.35 (1)	3.230 (4)	178 (4)

Symmetry codes: (ii) *x*−1, *y*, *z*; (iii) −*x*+1, *y*+1/2, −*z*+3/2; (iv) −*x*+2, *y*+1/2, −*z*+3/2.
